# Pregnancy during the pandemic: The psychological impact of COVID-19 on pregnant women in Greece

**DOI:** 10.18332/ejm/157463

**Published:** 2023-01-30

**Authors:** Athina Diamanti, Antigoni Sarantaki, Nafsika Kalamata, Victoria Vivilaki, Dimitra Varnakioti, Aikaterini Lykeridou

**Affiliations:** 1 Faculty of Health and Caring Sciences, Department of Midwifery, University of West Attica, Athens, Greece

**Keywords:** pregnancy, fear, STAI scale, perinatal mental health, COVID-19, maternal anxiety

## Abstract

**INTRODUCTION:**

The COVID-19 outbreak has affected the overall health of people worldwide. Historically, pandemics pose a challenge to psychological resilience, causing heightened stress levels. This study aimed to investigate the impact of the COVID-19 pandemic on the psychological state of pregnant women in Greece.

**METHODS:**

A survey study was conducted on a sample of 149 pregnant women in late 2020, including the ‘fear of COVID-19’ scale, a self-report instrument that assess fear of COVID-19 among the general population and the State-Trait Anxiety Inventory (STAI) scale which measures state and trait anxiety.

**RESULTS:**

Pregnant women with a mental health history tended to score higher on the ‘fear of COVID-19’ scale (mean ± SD: 19.48 ± 4.35) compared to pregnant women who had never had mental health problems before (17.12 ± 5.27). Moreover, pregnant women with anxiety as part of their personality tended to also score higher on the ‘fear of COVID-19’ scale. In all, 48.3% of pregnant women reported that their psychological state had been severely affected by the COVID-19 outbreak.

**CONCLUSIONS:**

Pregnant women were highly affected by the COVID-19 pandemic. A significantly increased ‘fear of COVID-19’ scale score was associated with self-reported pre-existence mental health conditions. Pregnant women with higher levels of ‘trait anxiety’ tended to report higher scores on the ‘fear of COVID-19’ scale.

## INTRODUCTION

The COVID-19 outbreak has affected the daily routine and overall health of people worldwide. Its high morbidity and mortality have also affected people’s mental health^1-3^. The most common response to any stressful situation – such as the COVID-19 pandemic – is an increase in overall stress. Pandemics seem to historically pose a challenge on peoples’ psychological resilience by causing heightened levels of stress^4^.

Pregnancy is considered to be a pleasant period; however, pregnant women are more likely to be at risk of increased anxiety since they undergo numerous physical and mental changes during the gestation period. The prevalence of anxiety disorder during pregnancy has been estimated to be 10% in developed countries and 25% in developing countries^1,5,6^. Pregnancy could also be affected by unpredicted factors such as the rise of a pandemic^7^.

Research indicates that pregnant women should be screened for anxiety and treated effectively before childbirth^8^. Therefore, maternal healthcare professionals need to recognize anxiety symptoms before pregnancy.

The COVID-19 pandemic has led to conflicting messages for health professionals and researchers, causing increased stress in pregnant women and in the general population, a condition which was actually expected. Initial studies regarding pregnancy outcomes suggested that maternal morbidity was similar to that of women of reproductive age and vertical transmission of the virus probably occurred in a small proportion of cases^7-11^. However, recent studies have demonstrated an increased risk of infection and eventual illness from the virus in pregnant women, rendering them consequently part of the high-risk groups^12-16^.

A more recent review and meta-analysis reported significantly elevated rates of antenatal depression and anxiety during the COVID-19 pandemic compared to norms that used a similar methodology before the COVID-19 era^17^. A study in Italy, on a sample of 100 pregnant women, reported that more than half of the participants rated the psychological impact of COVID-19 as severe, and two-thirds of the sample were found to be more anxious than usual^3^.

Based on the above indications, we hypothesized that the COVID-19 pandemic might also have a profound impact on pregnant women’s anxiety in Greece – considering that the base levels of anxiety and pregnancy characteristics in Greece, may be different. Thus, the aim of this study was to evaluate the psychological impact of the COVID-19 pandemic on pregnant women in Greece and its relationship with various pregnancy characteristics.

## METHODS

### Design

Data were collected between November and December 2020, the period of the ‘second wave’ of the maximum spread of COVID-19 in Greece. It was also the period of the second total lockdown imposed by the Greek government.

An anonymous online semi-structured questionnaire was developed using Google forms. The survey link was primarily shared via email, Facebook, and Instagram. It was also promoted through pregnancy-specific professional communities for distribution over their networks of pregnant women.

### Questionnaire

The questionnaire consisted of four sections. In section A, sociodemographic data were collected, including maternal age, education level, marital status, and employment status. In section Β, pregnancy history data were collected including gestational age, the number of previous pregnancies, type of birth in previous pregnancy, mental health issues, health issues in previous pregnancy etc. In section C, data were collected on participants’ awareness regarding the pandemic. In section D, two scales were included: 1) The 40 items State-Trait Anxiety Inventory (STAI), a validated scale for scoring anxiety^18^. STAI is a brief self-rated scale assessing state and trait anxiety. A value of ≥40 on the STAI is considered abnormal. The scale demonstrated strong psychometric properties on our sample, having a Cronbach alpha of 0.60 for the state and of 0.68 for the trait subscales, respectively; and 2) The Fear of COVID-19 Scale, a validated, seven-item scale^19^ assessing fear of COVID-19, has also demonstrated strong psychometric properties in our sample having a Cronbach alpha of 0.85.

### Ethics

Participants were informed about the purpose of the study, the fact that it would be anonymous and the terms of participation via a form that preceded the questionnaire, in which they had to give their written consent for their participation in the research. The conduct of this research also received approval from the committee of the midwifery department of the University of West Attica and was closely monitored during its execution by a three-member committee appointed by the assembly of the department.

### Statistical analysis

To investigate whether there was a correlation between the weeks of gestation and pregnant women’s fear of COVID-19, the Spearman’s rho correlation test was performed. Associations between scores of the ‘fear of COVID-19’scale and two groups were performed using Student’s t-test. Associations between pregnant women’s ‘state anxiety’ scale score, ‘trait anxiety’ scale score and their ‘fear of COVID-19’scale score, were examined by Spearman’s rho correlation test. Statistical analyses were conducted using ΙΒΜ SPSS statistical software.

## RESULTS

### Sample characteristics

The sample consisted of 149 pregnant women. The mean age of the participants was 31 ± 4.84 years, and their mean gestational age was 26.98 ± 9.53 weeks. The majority of the participants held a Bachelor’s degree (41.6%), and 28.9% of the sample had a postgraduate degree. Sociodemographic characteristics of the sample are presented in Table 1.

**Table 1:** Sociodemographic data of the included women, Greece, late 2020 (N=149)

*Characteristics*	*Mean ± SD (range)*
**Age** (years)	31.51 ± 4.84 (18–47)
**Gestational age** (weeks)	26.98 ± 9.53 (4–41)
***Education level***	***n (%)***
Primary school	1 (0.7)
Middle school – high school	15 (10.1)
Vocational training	25 (16.8)
University degree	62 (41.6)
Master of science	43 (28.9)
PhD	3 (2.0)
**Work status**	
Public sector	27 (18.1)
Private sector	69 (46.3)
Unemployed	25 (16.8)
Self-employed	19 (12.8)
Housewife	9 (6.0)
**Marital status**	
Single	6 (4.0)
Married	125 (83.9)
Cohabitation agreement	18 (12.1)
**Previous pregnancy**	
Yes	61 (40.9)
No	88 (59.1)

SD: standard deviation.

**Table 2:** Associations between ‘fear of COVID-19’ and physical and mental health problems among pregnant women in Greece during COVID-19, late 2020 (N=149)

*Problems during pregnancy*	*Mean ± SD*	*t*	*df*	*p*
**Physical**				
Yes	17.63 ± 5.53	-0.163	147	0.871
No	17.42 ± 5.17			
**Mental**				
Yes	19.48 ± 4.35	-1.944	147	0.50
No	17.11 ± 5.27			

### Sources of information

As far as the sources of information on COVID-19 during pregnancy and lactation were concerned, only 19% of pregnant women reported that the main source of information was an obstetrician (17%) or a midwife (2%), while the vast majority of them (81%) reported having received information from the public media (36.6%) or the Internet (44.4%).

**Figure 1:**
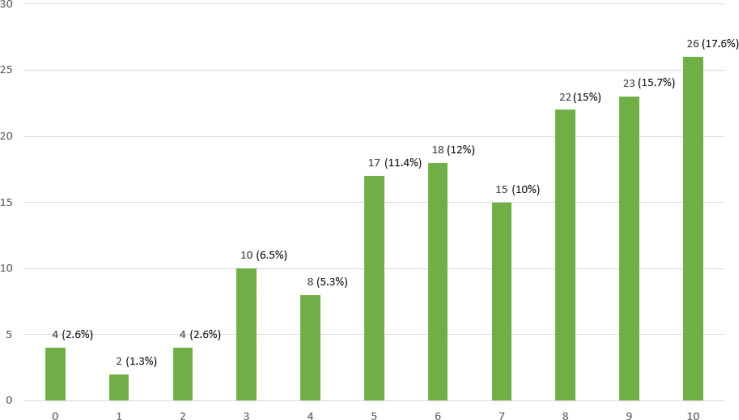
Likert scale results on ‘How much the psychology of pregnant women was affected by COVID-19 during their pregnancy’ (0: not at all, to 10: extremely)

Pregnant women were asked to rate whether their psychological state had been affected by COVID-19. A percentage of 48.3% reported that their psychological state had been severely affected, yet 33.4% of the participants reported that their psychological state was not affected so much (Figure 1).

Of the participants, 22.8% reported a ‘strong’ to ‘very strong’ concern about their health status, in case they were infected with COVID-19 during pregnancy. Moreover, 37.2% reported that what worried them most in the event of a SARS-CoV-2 infection, was the health of the fetus and their pregnancy outcomes.

Another major concern for pregnant women on their potential infection from COVID-19 was that they would not be accompanied by a close person while giving birth. Specifically, 76.5% of pregnant women reported being ‘very afraid’ to ‘severely afraid’ of giving birth without the presence of a close one. Only 16.4% did not seem to care so much if a close person would not be able to attend their labor.

### Associations between scores and traits

The participants’ mean score on the ‘fear of COVID-19’scale was 17.45 ± 5.20. Regarding the State-Trait Anxiety Inventory (STAI) scale, the participants score for the State was 47.60 ± 6.21 (range: 31–70) and that for the Trait was 46.11 ± 6.47 (range: 20–60). The results of Spearman’s rho correlation test showed that there was no statistically significant difference between the week of gestation and pregnant women’s fear of COVID-19 (rho=0.023, p=0.0782). Associations between scores of ‘fear of COVID-19’scale and nulliparous and multiparous pregnant women, suggested that there was no statistically significant difference in the scores of the scale between the two groups (17.31 vs 17.55; t=0.269, p=0.788).

No statistically significant difference was found between the scores of the ‘fear of COVID-19’ scale and women who had physical health problems during their pregnancy and those who had not (t = -0.163; p=0.871).

Finally, a statistically significant difference was found in the scores between women who had mental health problems in advance of their pregnancy and those who had not (t = -1.944; p<0.05). Specifically, pregnant women who previously had self-reported mental health issues reported a higher ‘fear of COVID-19’ scale score (19.48 ± 4.35) compared to those who had not (17.12 ± 5.27). There was no statistically significant difference between pregnant women’s ‘state anxiety’ scale score and their ‘fear of COVID-19’ scale score (rho=0.08; p=0.333).

In contrast, a statistically significant difference was found between pregnant women’s ‘trait anxiety’ scale score and their ‘fear of COVID-19’ scale score (rho=0.176; p=0.032). Specifically, pregnant women with higher levels of ‘trait anxiety’ tended to report higher scores on the ‘fear of COVID-19’ scale as well.

## DISCUSSION

Our study demonstrates how the COVID 19 pandemic has affected the psychological state and stress levels of pregnant women in Greece. More specifically, a large percentage of the participants reported that their psychological state had been severely affected by the COVID-19 pandemic. The presence of certain other aggravating factors associated with higher anxiety levels was investigated, such as having preexisting mental health problems.

According to our data, the COVID 19 pandemic induced a moderate increase in maternal anxiety as expressed by the mean ‘fear of COVID-19’ scale score of the participants. It was also found that a significantly increased ‘fear of COVID-19’ scale score was correlated with the pre-existence of mental health issues. Moreover, the results of the STAI scale scores were found to be quite high both for the trait and the state anxiety sub-scales. More specifically, pregnant women with high trait anxiety levels scored the highest on the ‘fear of COVID-19’ scale.

Furthermore, it was found that only 19% of the participant women received information from obstetricians and midwives, and their understanding of COVID-19 was acquired mainly from the internet and the public media. The joint percentage of 19% for women that received information from either their obstetrician or midwife is particularly small – thus worrisome – and contradicts the international literature^20^, where pregnant women are generally expecting to receive information concerning health issues primarily from the maternity healthcare providers who monitor their pregnancy. It is worth mentioning that a previous study suggested that when participants received information about health issues from the mass media, higher stress scores were reported^21^, in line with the results of our study.

Participants reported a high percentage of fear of abnormal perinatal outcomes and being highly worried about the consequences of a COVID-19 infection on their fetus’ health. Moreover, one of the major concerns for pregnant women was the possibility of giving birth unaccompanied by a close person due to a COVID-19 infection. Recent studies from America^22^ and Italy^1,3^ have reported an increase in pregnant women’s anxiety and fear levels^1,3,22^ due to the COVID-19 outbreak, which is definitely in accordance with our findings. In our study, however, we did not detect a statistically significant difference between the levels of ‘fear for COVID-19’ and the gestational age of the women, as observed in the previous studies^3,22^. More specifically, in the study of Moyer et al.^22^, women in the third trimester had higher stress scores, while the study from Italy showed that women’s psychological state and stress levels due to COVID-19 were more affected in the 1st trimester of pregnancy^3^. The findings of our study, however, were in accordance with the results of another study from Italy^1^ where a correlation between the gestational age and stress levels due to the COVID-19 outbreak was not found.

Our study indicated that pregnant women who had a mental health disturbance prior to pregnancy, reported higher ‘fear of the COVID-19’ scale scores than those who had not. This finding was in accordance with the results of a previous study which concluded that women with a mental health history were much more susceptible to high levels of stress, anxiety, and depression^23^. Additionally, in the study of Moyer et al.^22^, high-stress levels were more obvious in the 3rd trimester of pregnancy in women with a mental health history^22^.

Women’s mean score on the State Anxiety Inventory (STAI-S) scale was significantly higher than that previously reported^24^, a fact that emphasizes the effects of the COVID-19 pandemic on mental health. This was, however, similar to the findings of the Mappa et al.^1^ survey, which was also conducted in pregnant women during the COVID-19 pandemic. Moreover, the women’s mean score for the Trait Anxiety Inventory (STAI-T) scale was similar to the findings of Mappa et al.^1^, in which the median value of STAI-T score was 37 and in 38.2% of the study group the STAI-T score was ≥40. Additional previous studies reported that the lowest baseline mean STAI-S score in pregnancy was 35.6, a value that is close to the score of 40 which is considered as indicative of anxiety^25^.

### Limitations

The findings of this study must be interpreted in light of some limitations. Because online surveys are distributed to an unknown audience and voluntary participation can result in biased respondents selecting themselves into the sample, the results of this study may include a selection bias and are thus unlikely to be generalizable. Furthermore, because the majority of the participants were Caucasian women, the results may differ in other ethnic groups.

## CONCLUSIONS

To the best of our knowledge, this is the first study reporting on the psychological effects of COVID-19 in a sample consisting of pregnant women in Greece. It is worth mentioning that data were collected between the 25 November and 10 December 2020, which coincided with the second wave of COVID-19 outbreak in in Greece.

According to our findings, pregnant women have high levels of anxiety as well as ‘fear of COVID-19’ infection. The COVID-19 pandemic had a significant overall psychological impact on pregnant women. As a result, maternal healthcare providers must provide increased and consistent psychological support to pregnant women during this time. Finally, pregnant women with a mental health history or a high-risk pregnancy must receive early psychological interventions to avoid potential pregnancy stress-related complications.

## Data Availability

The data supporting this research are available from the authors on reasonable request.
